# Evaluating changes in negative posttrauma cognition as a mechanism of PTSD severity changes in two separate intensive treatment programs for veterans

**DOI:** 10.1186/s12888-022-04296-1

**Published:** 2022-11-04

**Authors:** Philip Held, Debra L. Kaysen, Dale L. Smith

**Affiliations:** 1 Department of Psychiatry and Behavioral Sciences, Rush University Medical Center, Chicago, IL USA; 2 Department of Psychiatry, Rush University Medical Center, 325 S. Paulina St., 2nd Floor, Chicago, IL 60612 USA; 3 Department of Psychiatry and Behavioral Sciences, Stanford University School of Medicine, Stanford, CA USA; 4 National Center for PTSD, VA Palo Alto Health Care System, Palo Alto, CA USA

**Keywords:** Negative posttrauma cognitions, Treatment mechanism, Intensive treatment, PTSD, Veterans

## Abstract

**Background:**

A wealth of evidence has illustrated that reductions in negative posttrauma cognitions (NPCs) predict improvement in posttraumatic stress disorder (PTSD) symptoms during treatment. Yet, the specific temporal arrangement of changes in these constructs is less well understood. This study examined the temporal association between NPC changes and PTSD symptom changes in two distinct intensive PTSD treatment samples.

**Methods:**

Data from 502 veterans who completed a 3-week CPT-based intensive PTSD treatment program was used to test the extent to which lagged NPC measurement predicted the next occurring PTSD severity measurement using linear mixed effects regression models. PTSD severity was assessed every other day during treatment. NPCs were assessed at three treatment timepoints. A second sample of 229 veterans who completed a 2-week CPT-based intensive PTSD treatment program was used to replicate these findings.

**Results:**

Across both intensive PTSD treatment programs, NPCs generally increased from intake the end of the first treatment week, which was followed by gradual decreases in NPCs throughout the rest of both programs. Change in NPCs during both the 3-week (*b* = .21, *p* < .001, *R*^2^ = .38) and the 2-week programs (*b* = 0.20, *p* < .001, *R*^2^ = .24) were significant predictors of change in PTSD symptom severity. However, the reverse was true as well, with change in PTSD severity predicting latter change in NPCs during both the 3-week (*b* = 1.51, *p* < .001, *R*^2^ = .37) and 2-week (*b* = 1.37, *p* < .001, *R*^2^ = .33) programs, further raising questions about temporality of the association between NPCs and PTSD symptom severity during treatment.

**Conclusions:**

The present study demonstrated that changes in NPCs may not temporally precede changes in PTSD symptom severity in PTSD treatment samples. Instead, we observed earlier PTSD symptom changes and a bidirectional association between the two constructs across both samples. Clinically, the study supports the continued focus on NPCs as an important treatment target as they are an important indicator of successful PTSD treatment, even if they may not be a direct mechanism of treatment-based changes in PTSD severity. Future research should attempt to identify alternative mechanisms of change in CPT.

**Supplementary Information:**

The online version contains supplementary material available at 10.1186/s12888-022-04296-1.

Posttraumatic stress disorder is associated with changes in thinking content and processes, where trauma-related blame may be misattributed or beliefs about self, others, or the world may become inaccurate or too extreme [1]. Negative posttrauma cognitions (NPCs) are thought to develop following trauma exposure when trauma-related information cannot be adaptively processed [2–4]. NPCs are theorized to interfere with the successful recovery from traumatic experiences [2–4] and their development following trauma, or existence prior to trauma, may increase the risk for individuals to develop PTSD [5–8]. For this reason, NPCs have become a hypothesized mechanism of change and treatment target in various evidence-based psychotherapies for PTSD [4, 9–11]. Indeed, failure to engage NPCs has been posited as a contributor to suboptimal treatment outcomes [12].

Primarily cognitive PTSD treatments, such as Cognitive Processing Therapy (CPT) [4], aim to directly change cognitions through therapeutic techniques, such as cognitive restructuring [13]. In these treatments, individuals are taught to evaluate their NPCs against evidence related to the trauma and develop alternative, more adaptive ways of processing the experience. Reducing the strength of NPCs and generating plausible alternative beliefs is thought to allow for adaptive information processing and ultimately result in a reduction in PTSD symptoms [4]. Successful PTSD treatment has repeatedly been shown to reduce NPCs across populations [14–22]. Moreover, NPCs have been implicated in the long-term maintenance of gains, with less strongly endorsed NPCs being associated with greater improvement in PTSD symptom severity up to 10 years after treatment [23]. Similar findings have been observed in intensive PTSD treatments, in which evidence-based treatments are delivered daily or multiple times per day over the course of 1 to 3 weeks. Despite their brevity, studies on intensive PTSD treatments suggest that individuals experience large NPC changes and that these changes are predictive of short-term PTSD symptom reduction over the course of treatment [24] as well as of maintenance of gains for up to a 1 year following treatment completion [25].

Although a wealth of evidence has illustrated that reductions in NPCs predict improvement in PTSD symptoms during treatment [17, 20, 22, 24, 26, 27], the specific temporal arrangement with regards to changes in NPCs and changes in PTSD symptoms is less well understood [28]. Only a few studies to date have been designed to effectively examine the temporal association between NPCs and PTSD severity, although most are limited by relatively small sample sizes, resulting in findings not replicating across samples (for a review, see [9, 28]). Consequently, researchers have called for more rigorous evaluations of NPCs as treatment mechanisms [9]. Recent studies have demonstrated a bi-directional relationship between NPC and PTSD symptom changes [28, 29]. Specifically, a recent study by Lee et al. [28] indicated that changes in NPCs and PTSD severity may actually occur concurrently. The study’s findings suggested the presence of a bidirectional relationship between these variables over time in two distinct PTSD treatments, including CPT, that were delivered weekly as part of clinical trials with regular follow-ups to 60 weeks following the first treatment sessions [28]. Such findings raise questions about the role of NPC changes as a mechanism of treatment-based PTSD severity change in the traditional sense; for negative cognitions to effectively serve as a mechanism of PTSD change NPC changes would be expected to precede improvement in PTSD severity [30]. Replication of mechanistic findings is also a criterion for establishing a mediator [30]. Given the potential implications of such findings for the field’s understanding of NPCs as a mechanism of PTSD symptom change during treatment, it is critical that these recent findings by Lee et al. [28] are replicated in different samples and treatment approaches. Moreover, it is important to extend existing research by examining the temporal association between NPCs and PTSD severity changes in novel intensive PTSD treatments to determine whether the condensed nature of this delivery format further impacts the association between NPC and PTSD severity changes. Much of the research examining NPC change as a PTSD treatment mechanism has been tested in controlled clinical trials [16, 17, 22, 27–29]. It is important to extend this research into clinical programs where inclusion criteria are often broader compared to efficacy trials and intervention delivery is not as closely monitored. Both of these factors can dilute any effects observed in highly controlled trials, highlighting the importance to examine the generalizability of findings surrounding NPC as a mechanism outside of efficacy research, as only relatively few studies to date have done [20]. Finally, better understanding of the role of NPC in treatment and the temporal associations between NPC and PTSD symptom change is clinically important. Understanding how and when NPC’s change and how this is related to change in PTSD help evaluate NPC’s role in cognitive treatments. Moreover, in cognitive therapies clinicians may note change or a lack of change in cognitions in session or with practice assignments, even if they are not formally assessing NPC’s. Often change in cognition is viewed as a positive prognostic indicator. However, if cognitive change is not a core mechanism, then clinician behavior may need to shift to address other potential treatment elements.

The goal of the present study was to examine the temporal association between NPC changes and PTSD symptom changes in CPT-based intensive PTSD treatments delivered as part of clinical care as opposed to clinical trials and to evaluate the impact changes in negative posttrauma cognitions have on intensive PTSD treatment outcomes. In light of the inconsistent findings around NPC change as a potential mechanism of PTSD symptom change and recent findings by Lee et al. [28] suggesting that PTSD and NPC are correlates rather than NPC change acting as a true mechanism, we wanted to replicate Lee et al.’s [28] study in a larger sample of veterans receiving CPT as part of a 3-week intensive PTSD treatment and extend the analyses using linear mixed effect regression models to examine the extent to which lagged NPC measurement predicted the next occurring PTSD severity measurement. We also set out to replicate our results internally using a separate 2-week CPT-based intensive PTSD treatment dataset to increase the generalizability of our findings. In line with precision medicine approaches, we believed that it would be important to examine post hoc mediation in a subset of participants who showed an initial rapid reduction in NPCs as this could indicate the possibility of NPCs mediating changes in PTSD severity in some, but not all, individuals. To test this assumption, we planned to examine a subset of individuals who exhibit NPC improvements early in treatment, even if temporal precedence may not exist in the average effect across all participants.

## Method

### Participants

The present study used data from 502 veterans who completed a 3-week CPT-based intensive PTSD treatment program between April 2016 and March 2020. Veterans in the 3-week ITP were on average 41.35 years old (SD = 9.43), and the majority identified as male (65.94%), White (67.33%), and not Latinx (79.88%).

A second sample of 229 veterans who completed a 2-week CPT-based intensive PTSD treatment program between June 2020 and January 2022 was used to internally replicate the study. Veterans in the 2-week ITP were on average 42.71 years old (SD = 9.02), and the majority identified as female (51.98%), White (63.88%), and not Latinx (82.38%). Additional sample characteristics are displayed in Table 1.

**Table 1:** Demographic characteristics by intensive PTSD treatment program

	2-Week ITP (*N* = 229)		3-Week ITP (*N* = 502)	
	*N*	%	*N*	%
Sex				
Male	109	48.02	331	65.94
Female	118	51.98	171	34.06
Ethnicity				
Not Latinx	187	82.38	401	79.88
Race				
American Indian/ Alaskan Native	4	1.76	10	1.99
Asian	5	2.20	6	1.20
Black or African American	50	22.03	101	20.12
Native Hawaiian/ Pacific Islander	4	1.76	3	0.60
Other	16	7.93	42	8.37
Refusal	1	0.44	1	0.20
White	145	63.88	388	67.33
Military Service Branch				
Air Force	27	11.84	42	8.37
Army	117	51.32	333	66.33
Coast Guard	2	0.88	4	0.80
Marines	50	21.93	75	14.94
Navy	32	14.04	48	9.56
Service Era				
Post September 11, 2001	195	86.28	451	89.84
Deployed				
Yes	163	71.49	395	78.69
	*M*	*SD*	*M*	*SD*
Age	42.71	9.02	41.35	9.43

*ITP* Intensive PTSD Treatment Program

The acceptance process for the 3- and 2-week intensive PTSD treatments was identical. In order to be eligible for treatment, veterans needed to meet the diagnostic criteria for PTSD assessed via the Clinician Administered PTSD Scale for DSM-5 [31]. Veterans throughout the United States were eligible for treatment. All transportation to and from the treatment program, lodging in a nearby hotel, and treatment itself were provided at no cost. Veterans were ineligible for the intensive PTSD treatment programs if they had unstable housing, inability to independently complete activities of daily living, a suicide attempt in the last 30-days, untreated psychosis or mania, or alcohol or other drug dependence. Veterans were encouraged to discontinue the use of alcohol or drugs during treatment, but alcohol and drug use during treatment was not formally monitored.

### Procedures

Both programs were built around CPT. In the 3-week intensive PTSD treatment program, veterans received 14 daily individual CPT sessions and 13 daily group CPT sessions. In addition, veterans participated in daily psychoeducation, skill building, yoga, and mindfulness groups. In the 3-week program, veterans received a total of 104 hours of clinical programming. In the 2-week program, veterans received 16 individual CPT sessions that were delivered twice daily. In addition, veterans participated in daily skill building and yoga or mindfulness groups. In the 2-week program, veterans received a total of 67 hours of clinical programming. Both the 3- and 2-week intensive PTSD treatment programs have been described in additional detail elsewhere and been shown to provide equivalent results despite the differences in treatment length and programming [32].

The study procedures were approved by the Institutional Review Board at Rush University Medical Center with a waiver of consent as all assessments were collected as a part of routine care.

### Measures

#### Demographic characteristics

As part of the intake evaluation, participants reported various demographic and military specific characteristics. These included age, sex, ethnicity, race, military service, branch, whether they served before or after September 11, 2001, and whether they deployed. The variables were used to characterize the sample.

#### Negative posttrauma cognitions

NPCs were assessed using the Posttraumatic Cognitions Inventory (PTCI) [33], a 33-item self-report measure. The PTCI possible score range is 33 to 231 with higher scores indicating stronger negative beliefs. During the 2-week ITP, this measure was given at intake and on days 4 and 9. In the 3-week ITP, the PTCI was given at intake and on days 4, 9, and 14. The PTCI was assessed in the morning prior to any treatment sessions that day. Cronbach’s alpha ranged from .951-.982 in the 3-week and from .953-.970 in the 2-week ITP.

#### PTSD symptom severity

PTSD symptom severity was assessed using the PTSD Checklist for DSM-5 (PCL-5) [34], a 20-item self-report measure based on the DSM-5 diagnostic criteria. The PCL-5 possible scores range is 0 to 80 with higher scores indicating more severe PTSD symptoms. At intake, veterans reported their symptoms for the past month. During treatment, past week severity was reported. In the 2-week program, the PCL-5 was administered at intake and on days 1, 3, 5, 6, 8, and 10. In the 3-week program, the PCL-5 was administered at intake and on days 2, 3, 5, 6, 8, 10, 11, 13, and 14. The PCL-5 was assessed in the morning prior to any treatment sessions that day. Cronbach’s alpha ranged from .888-.962 in the 3-week and from .918-.943 in the 2-week ITP.

### Statistical analysis

To replicate parts of Lee et al.’s [28] analytical design, we initially examined the overall timing of changes in NPCs and PTSD severity from intake using standardized mean gain scores (ESsg; [35]). This metric indicates the standardized change from intake in each measure and can be interpreted as a standardized effect size for longitudinal change much like other variants of Cohen’s *d* that account for repeated measures [36]. By comparing the timelines of change in both NPCs and PTSD symptom severity we were able to descriptively determine which improved first on average across all participants, though this did not provide specific information about these relationships within participants.

Next, we examined the nature of the relationship between NPCs and PTSD severity through linear mixed effect regression models (LMM), often viewed as a gold-standard for longitudinal research analysis. The linear mixed effects model is inherently flexible regarding measurement timepoints, variability, and variances/covariances over time, and accommodates both differing timepoint measurements across participants and missingness in outcome data over time as well [37]. Although NPCs have been repeatedly and definitively demonstrated to be a time-varying predictor of PTSD improvements in longitudinal analyses of CPT both within-subjects and between groups using LMMs [23–25], demonstrations that changes in NPCs predict PTSD severity changes over time may not fully elucidate the temporality of this relationship. A more intensive examination of the temporal relationship between these two variables using LMMs in a sample of this size has not been explored to our knowledge. We utilized an approach previously suggested to elucidate the temporal relationship using LMMs [38]. This approach involved partitioning the within-subjects and between-subjects variability in NPCs and exploring both as lagged predictors of PTSD symptom severity. Following partitioning within- and between-subject variation in NPCs, we examined the extent to which lagged NPC measurement predicted the next occurring PTSD severity measurement. For example, in the present study intake PTCI was used to predict program Day 2 PCL-5, Day 4 PTCI was used to predict Day 5 PCL-5, Day 9 PTCI was used to predict Day 10 PCL-5, etc. We also explored PTCI by time interactions to determine whether the relationship between NPCs and PTSD severity changed over time. To address the potential for bidirectional relationships, we also examined models in which the same procedure was followed but using PTSD severity measurement to predict the next occurring NPC measurement. For example, Day 3 PCL-5 was used to predict Day 4 PTCI, etc. Finally, to explore the possibility of NPCs mediating changes in PTSD severity in some, but not all, veterans, we repeated these analyses on only a subset of individuals in the larger 3-week program dataset who improved in PTCI during the first week of the intensive treatment program (e.g., by Day 4). These analyses were not conducted in the 2-week program due to the smaller sample size and the resulting relatively small number of individuals who reported PTCI improvement at the time of the first treatment measurement. The purpose of this step was to elucidate whether the temporal relationships between PTCI and PCL-5 were similar among these individuals as with the larger sample, which also included individuals who did not experience such reduction during the first week. All models described above adjusted for age and sex, and effect sizes for LMMs were obtained via Edwards et al. [39] variant of *R*^2^ for LMMs. To avoid potential overlap between the cognition items on the PCL-5 (items 9 & 10) and the PTCI, we also conducted the aforementioned analyses without these two items. As the results did not substantially differ, we decided to only present the results using the full PCL-5 below (see supplemental Table S1 for results without these items). Linear mixed effects models were examined in Stata version 17 [40], and figures utilized R version 4.1.2 [41].

## Results

Improvements in NPCs were moderate to large, with those in the 3-week program improving by 30.19 points on the PTCI (SD = 44.78; ESsg = 0.71), and those in the 2-week program improving by an average of 22.68 points (SD =38.06; Essg = 0.59). Similarly, improvements in PTSD symptom severity were large, as veterans in the 3-week program improved by 21.43 points on the PCL-5 (SD = 18.40; Essg = 1.31), and veterans in the 2-week program improved by an average of 17.74 points on the PCL-5 (SD = 15.63; Essg = 1.21).

**Fig. 1:**
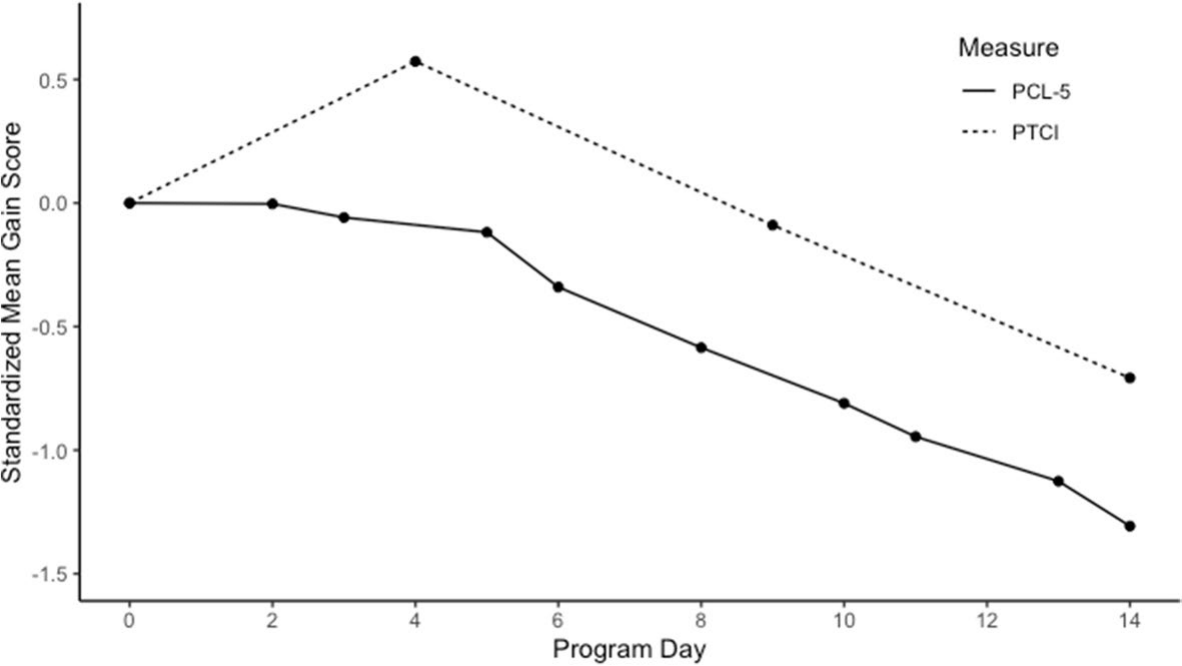
Temporal pattern of change in PCL-5 and PTCI in the 3-week ITP. *Note:* ITP = Intensive PTSD Treatment Program. PTCI = Posttrauma Cognitions Inventory. PCL-5 = PTSD Checklist for DSM-5

**Fig. 2:**
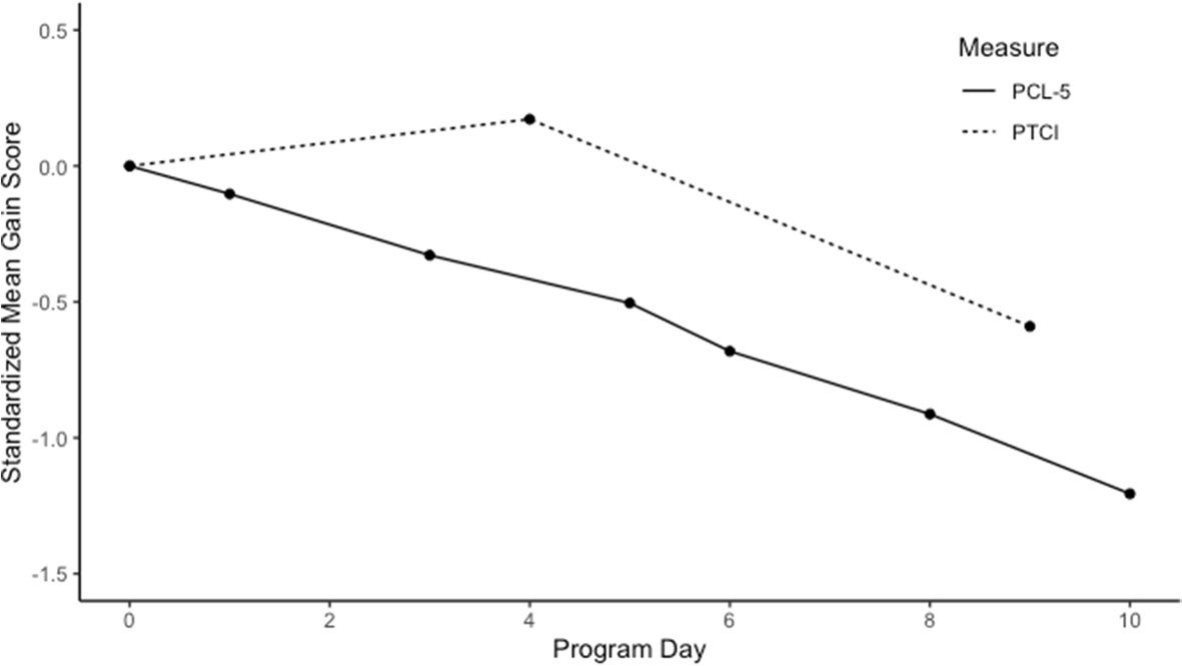
Temporal pattern of change in PCL-5 and PTCI in the 2-week ITP. *Note:* ITP = Intensive PTSD Treatment Program. PTCI = Posttrauma Cognitions Inventory. PCL-5 = PTSD Checklist for DSM-5

Across both intensive PTSD treatment programs, NPCs generally increased from intake the end of the first treatment week, which was followed by gradual decreases in NPCs throughout the rest of both programs (see Figs. 1 and 2 and supplemental Figs. S1 & S2 for results with PTCI subscales).^1^ Unlike NPCs, PTSD severity generally decreased steadily throughout both early and later parts of both programs. Thus, the temporal order of improvement suggests that on average PTSD symptoms begin to decrease early during the program, while improvement in NPCs do not generally occur until later, typically after the first week of treatment.

Linear mixed effects regression models were conducted to examine the relationship between negative cognitions and PTSD over time.^2^ Results indicated that unstructured covariance was preferred, based on information criteria, and that both linear and quadratic time were significant (see Table 2). Change in NPCs during both the 3-week (*b* = .21, *p* < .001, *R*^2^ = .38) and the 2-week programs (*b* = 0.20, *p* < .001, *R*^2^ = .24) were significant predictors of change in PTSD symptom severity. Partitioning of between and within-subjects effects of PTCI indicated that both were significant predictors of PTSD severity in both programs (see Table 2). Thus, within-subject variation in NPCs predicted individual change in PTSD symptom severity. This association was also apparent between subjects. The time by PTCI interaction was also significant in the 3-week program (*p* < .001), but not the 2-week program (*p* = .736), indicating that the strength of the relationship between NPCs and PTSD severity changed during the course of the program. This appears to have been driven by the strength of the relationship between NPCs and PTSD symptom severity increasing over the course of treatment in the 3-week program, as correlations between PTCI and PCL-5 were *r* = .42 during the first week but increased to *r* = .84 by the end of the 3-week program. This increase was less apparent in the 2-week program, with correlations between measurement ranging from *r* = .64 during the first week and *r* = .72 at the end of the program.

**Table 2:** Models predicting PCL-5

Predictor	3-week ITP		2-week ITP	
	*b* (95% CI)	*p*	*b* (95% CI)	*p*
Time	−2.64 (−3.35, −1.93)	< .001	−3.17 (−3.99, −2.36)	< .001
Time^2^	0.10 (0.04, 0.15)	.001	0.16 (0.18, 0.23)	< .001
Age	0.08 (−0.01, 0.17)	.051	0.05 (−0.12, 0.23)	.544
Sex	0.13 (−2.56, 2.83)	.922	−2.70 (−5.95, 0.56)	.105
PTCI	0.21 (0.19, 0.23)	< .001	0.20 (0.16, 0.23)	< .001
PTCI x Time	0.02 (0.01, 0.02)	< .001	< .001 (<.001)	.736
PTCI within^a^	0.16 (0.14, 0.18)	< .001	0.11 (0.07, 0.15)	< .001
PTCI between^a^	0.27 (0.25, 0.30)	< .001	0.30 (0.26, 0.35)	< .001

*ITP* Intensive PTSD Treatment Program, *PCL-5* PTSD Checklist for DSM-5, *PTCI* Posttraumatic Cognitions Inventory

^a^Partitioned between and within-subjects effects of PTCI were examined in models without overall PTCI, due to overlap between these variables

We next examined the potential for a bidirectional relationship using LMMs predicting PTCI with lagged PCL-5 measurements. Results indicated that PTSD symptom severity significantly predicted subsequent NPCs in both the 3-week (*b* = 1.51, *p* < .001, *R*^2^ = .37) and 2-week (*b* = 1.37, *p* < .001, *R*^2^ = .33) programs (see Table 3). Similar to models predicting PTSD severity, this was true for both between-subject and within-subject variability in NPC severity. Thus, although changes in NPCs predicted subsequent PTSD severity as described above, the reverse is also true. Changes in PTSD severity predicted subsequent negative cognitions both within-subjects and between subjects. Additionally, the proportion of variability in PTCI over time that cross-lagged PCL-5 can account for was roughly the same as seen in models using PTCI to predict PCL-5 across both the 3- and 2-week programs.

**Table 3:** Models predicting PTCI

Predictor	3-week ITP		2-week ITP	
	*b* (95% CI)	*p*	*b* (95% CI)	*p*
Time	4.54 (3.77, 5.33)	< .001	7.36 (5.67, 9.06)	< .001
Time^2^	−0.39 (−0.45, −0.33)	< .001	−0.95 (−1.14, − 0.75)	< .001
Age	− 0.21 (− 0.43, 0.02)	.068	0.01 (− 0.37, 0.38)	.968
Sex	2.77 (−4.31, 9.84)	.444	5.18 (−1.65, 12.03)	.137
PCL-5	1.51 (1.41, 1.61)	< .001	1.37 (1.20, 1.55)	< .001
PCL-5 x Time	0.04 (0.03, 0.06)	< .001	0.03 (−0.01, 0.07)	.083
PCL-5 within^a^	1.20 (1.08, 1.32)	< .001	1.07 (0.83, 1.32)	< .001
PCL-5 between	2.04 (1.89, 2.19)	< .001	1.63 (1.39, 1.86)	< .001

*ITP* Intensive PTSD Treatment Program, *PTCI* Posttraumatic Cognitions Inventory, *PCL-5* PTSD Checklist for DSM-5

^a^Partitioned between and within-subjects effects of PTCI were examined in models without overall PTCI, due to overlap between these variables

**Fig. 3:**
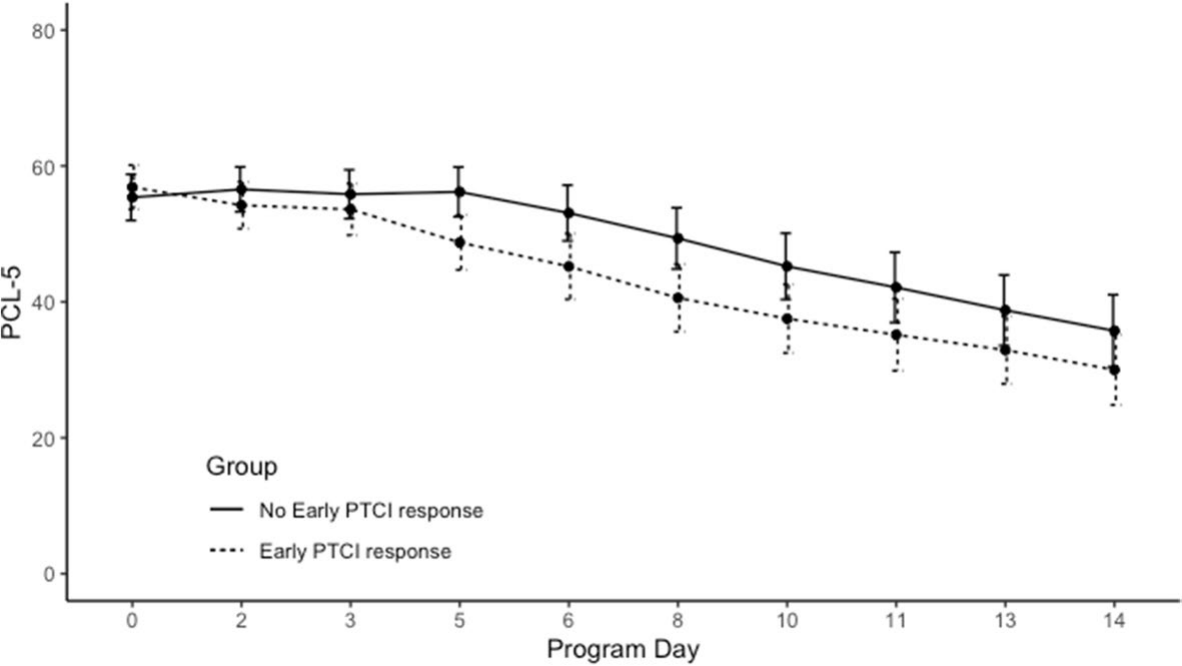
Difference in PTSD severity trends over time based on whether participants decreased in negative posttrauma cognitions early in the 3-week ITP. *Note:* ITP = Intensive PTSD Treatment Program. PTCI = Posttrauma Cognitions Inventory. PCL-5 = PTSD Checklist for DSM-5. Error bars represent 95% CIs

One of our goals was to explore whether NPCs acted as a mechanism for only a subset of veterans. In the 3-week program, total of 116 veterans (24%) improved in PTCI by the end of the first week. These individuals had 7.48 points lower mean PCL-5 scores at the end of the first week, significantly lower overall PCL-5 scores over time (*p* = .016), as well as greater improvement in PTSD severity (*p* = .001) than those who did not improve in NPCs during the first week (see Fig. 3). Among these individuals with early PTCI improvement, LMMs still indicated that changes in NPCs were significant predictors of changes in PTSD symptom severity, and that this was significant for both within-subject and between-subject variation in PTCI^3^ (see Table 3). However, similar to the full sample, the bidirectional relationship was also clear. In models predicting NPCs using preceding session measurement of PTSD severity, PTSD severity remained a significant predictor of NPCs. This was true of both between- and within-subject changes in PTSD severity. This indicates that although early treatment improvement in NPCs may occur in these individuals, and these individuals also correspondingly improve more in PTSD severity, the relationship between NPCs and PTSD symptom severity over the course of the program still appears to be a bidirectional one among these participants.

## Discussion

The present study demonstrated that changes in NPCs did not temporally precede changes in PTSD symptom severity in the two distinct intensive PTSD treatment samples. Instead, we observed earlier PTSD symptom changes and a bidirectional association between the two constructs across both samples. In other words, although NPC changes predicted changes in subsequent PTSD severity, the reverse was also true. As such, the results lend further support that NPC changes may not be a mechanism, or may not be the only mechanism through which PTSD symptoms change in treatment. Instead, as previously suggested [28], it appears that NPCs are a related construct which changes alongside PTSD symptoms. This proposition fits with the DSM-5 criteria for PTSD [1], which conceptualizes NPCs as one of many symptoms of PTSD. NPCs can therefore still be considered to be an important marker of PTSD treatment response and a treatment target, even if they do not play the previously hypothesized mechanistic role. Moreover, these findings have important implications for theories of cognitive therapies for PTSD and suggest that mechanisms other than, or in addition to, cognitive change should be explored. This could include incorporating the role of inhibitory learning, or emotion regulation as other potential mechanisms of action for treatment [42, 43].

Across both CPT-based intensive PTSD treatment programs examined in this study, NPCs initially increased until approximately the end of the first week of treatment, whereas average PTSD symptom severity decreased steadily during the same timeframe. Based on prior research on weekly PTSD treatments [16, 18, 19, 28], we did not expect to observe an increase in NPCs. These findings were especially surprising since veterans had completed a substantial number of individual CPT sessions by the time PTCI was measured on Day 4 and individuals generally tend to report NPC reductions by this time during the course of CPT [16, 18, 19, 28]. Although we have no way of conclusively determining why NPCs may increase from intake to the end of the first week in two distinct intensive treatments with notably different structures, it is possible that the intensive nature of treatment increases individuals’ awareness of their NPCs. In the ITPs, individuals received multiple CPT sessions per day, likely resulting in increased awareness of their NPCs that may be reflected via stronger endorsement on the PTCI. Future research on other ITPs, especially other CPT-based ITPs, is needed to evaluate whether this is a common phenomenon in ITPs. Another possibility is that the program structure, which included mindfulness, may have led individuals to become more aware of their cognitions and thus endorse them more strongly on the PTCI. Lastly, it is also possible that this is related to the infrequent measurement of NPCs in the present samples. In both programs, NPCs were assessed at intake before starting treatment when individuals may not yet be as aware of their NPCs. At the time of the next NPC measurement on Day 4, individuals had already completed several CPT sessions, which specifically work on identification of NPCs [4], and were thus more likely to recognize NPCs. Thus, although it appears as though veterans’ NPCs worsened, this may have been an artifact of measurement timing or increased awareness. Instead, it is likely that individuals simply became more aware of their NPCs during the early phases of treatment compared to intake, which is one of the main goals of CPT [4]. Additional research utilizing more frequent measurements of NPCs in intensive PTSD treatments is needed in order to test the aforementioned hypothesis. Regardless of these differences between our findings and those previously reported by Lee et al. [28], the overall results regarding the lack of mediation in the traditional sense [30] were largely replicated.

One specific goal of this study was to explore whether the temporal precedence may be observable among a subset of veterans who exhibited initial changes in NPCs in the 3-week program. We initially hypothesized that even though NPC change may not be a mechanism for everyone, it may be a mechanism for some individuals. However, even among veterans who reported early NPC reductions there was no clear temporal precedence of NPC changes to PTSD symptom severity changes. However, veterans who reported early NPC changes also reported greater PTSD symptom reductions by the end of the first week compared to those who did not report early NPC reduction. The comparatively greater PTSD symptom reduction among veterans who reported early NPC changes were maintained over the course of the entire 3-week program, further highlighting the interconnection between these two constructs. The present findings suggest that achieving more rapid NPC reductions would likely be associated with quicker PTSD symptom improvements. Thus, identifying strategies to reduce NPCs as early as possible in intensive treatments may help further improve PTSD treatment outcomes, even if NPC changes do not function as a true mechanism.

A clear limitation of the current study are the relatively infrequent measurement timepoints for both the PCL-5 and the PTCI. Although the PCL-5 was assessed every other day during treatment, this timeframe encompasses as many as four CPT sessions in the 2-week program. Similarly, the PTCI was only measured before the program and two and three times during the 2- and 3-week programs, respectively. This infrequent measurement of NPCs prevents us from being able to detect potential nuances in NPC change. Additionally, despite the repeated measurement throughout treatment and the clear differences in PTSD and NPC change patterns observed in this study, the non-simultaneous assessment of both the PCL-5 and PTCI is also not ideal to evaluate the temporal order of changes that occurred. The different timeframes of both measures are another important limitation to consider. Whereas the PCL-5 asked for veterans to rate symptoms based on the past week during treatment, the PTCI does not ask for a specific timeframe, thus potentially being more reflective of momentary changes compared to more steady, “averaged” reports of past week PTSD symptoms on the PCL-5. Thus the PTCI may reflect more momentary changes compared to more steady, “averaged” reports of past week PTSD symptoms on the PCL-5. Future studies should use ecological momentary assessment designs to better examine how state-like shifts in cognitions and symptoms predict each other to better determine how cognitions may function during treatment. Additionally, the analytic approach using LMMs created two separate models to examine bidirectional effects, rather than one as often examined in cross-lagged panel designs. However, we believe that ability to model random effects structure * appropriately within LMMs without making * assumptions inherent to the cross-lagged panel approach was important here. Finally, the intensive PTSD treatments in this study combined CPT with additional interventions, such as mindfulness, where veterans were encouraged to become aware of their thoughts, among others, which may have increased their awareness of their NPCs compared to what would be the norm for standalone evidence-based PTSD treatments. Thus, findings from the present study may not necessarily generalize to evidence-based PTSD treatments that are delivered as standalone interventions outside of an intensive PTSD treatment program.

Despite these limitations, the present study provided additional support for NPC changes being a correlate of PTSD symptom severity changes rather than a mechanism that temporally precedes changes in two separate intensive PTSD treatment programs and thus extended findings previously demonstrated for weekly PTSD treatments [28]. As a clear strength of the present study, the findings from the CPT-based 3-week ITP were replicated in second CPT-based program that different in programming length and content (i.e., 2-week ITP). Going forward, it will be important to evaluate how to change NPCs more quickly and effectively to further improve PTSD treatment, even if they are not a mechanism in the truest sense. Future research on NPCs could also benefit from examining NPCs in a more individualized form. For example, evaluating individuals’ specific NPCs they have developed in response to traumatic experiences, rather than asking them to evaluate cognitions trauma survivors commonly endorse on a broad measure such as the PTCI may also improve our understanding of the association between person-specific NPCs and PTSD symptom severity. Further, combining such individualized approaches with more frequent measurements of both NPCs and PTSD severity, such as via ecological momentary assessments, may provide additional insights. For example, it is possible that individuals’ specific trauma cognitions change before they notice reductions in their PTSD symptoms, even if they may not report NPC changes on generally applicable measures, such as the PTCI until later in treatment (e.g., individuals may inform their therapists of changes in their thinking about their trauma but continue to fill out the PTCI as they had in previous sessions). Additional research is needed to identify true PTSD treatment mechanisms. Clinically, the present findings support the continued focus on NPCs as an important treatment target. Specifically, future research should evaluate how NPCs may be changed more quickly as this would have positive impacts on overall PTSD symptom severity reductions. Finally, to further elucidate the role of NPC change in PTSD treatment, it will be important to examine whether the findings presented here can be replicated in PTSD treatments that do not have a specific focus on NPCs (e.g., Prolonged Exposure and Eye Movement Desensitization and Reprocessing).

## Supplementary Information

**Additional file 1: Table S1.** Models Predicting PCL-5 without items 9 and 10. **Figure S1.** Time trends for PTCI subscales and PCL-5 in 3-week program. **Figure S2.** Time trends for PTCI subscales and PCL-5 in 2-week program.

## Data Availability

The datasets used in the current study are not publicly available. Datasets can be obtained from the corresponding author upon reasonable request.

## Footnotes

1 We also examined the temporal relationship between each PTCI subscale and PTSD severity over time. Results were nearly identical to those using the full scale, so they were not reported here.

2 We also explored parallel process latent growth curve analysis in MPlus version 8, to fully replicate Lee et al. [28]. Results aligned with Lee et al. [28], with positive relationships between changes in PTCI and PCL-5 indicating that those with greater changes in NPCs had greater changes in PTSD symptom severity over the course of the intensive PTSD treatment program. Because these analyses generally overlap with those presented here using LMMs, and were primarily conducted to ensure replication, they are not reported here in detail.

3 Quadratic time was not significant in this reduced sample and was thus not included in final models.
