# Supplementary material for: Evaluating changes in negative posttrauma cognition as a mechanism of PTSD severity changes in two separate intensive treatment programs for veterans
Source: BMC Psychiatry. 2022 Nov 4;22:683. doi: 10.1186/s12888-022-04296-1 (PMC9635118; doi:10.1186/s12888-022-04296-1)

**Supplemental Materials**

Table S1. Models Predicting PCL-5 without items 9 and 10

|  | 3-week program | | | 2-week program | | |
| --- | --- | --- | --- | --- | --- | --- |
| Predictor | *b* (95% CI) | *p* | *b* (95% CI) | | *p* |  |
| Time | -0.37 (-0.55, -0.18) | < .001 | -1.33 (-1.70, -0.97) | | < .001 |  |
| Time^2^ | -0.08 (-0.09, -0.07) | < .001 | -0.03 (-0.06, 0.00) | | .086 |  |
| Age | 0.05 (-0.02, 0.13) | .167 | 0.05 (-0.11, 0.20) | | .550 |  |
| Sex | -0.19 (-1.74, 1.35) | .806 | -2.23 (-5.10, 0.65) | | .130 |  |
| PTCI | 0.11 (0.10, 0.11) | < .001 | 0.17 (0.14, 0.20) | | < .001 |  |
| PTCI within^a^ | 0.08 (0.07, 0.09) | < .001 | 0.11 (0.07, 0.15) | | < .001 |  |
| PTCI between^a^ | 0.23 (0.21, 0.25) | < .001 | 0.30 (0.26, 0.35) | | < .001 |  |
| PTCI x Time | 0.02 (0.01, 0.02) | < .001 | .003 (<-0.01, 0.01) | | .191 |  |

Notes: PCL-5 = PTSD Checklist for DSM-5. PTCI = Posttraumatic Cognitions Inventory. ^a^Partitioned between and within-subjects effects of PTCI were examined in models without overall PTCI, due to overlap between these variables.

Figure S1. Time trends for PTCI subscales and PCL-5 in 3-week program


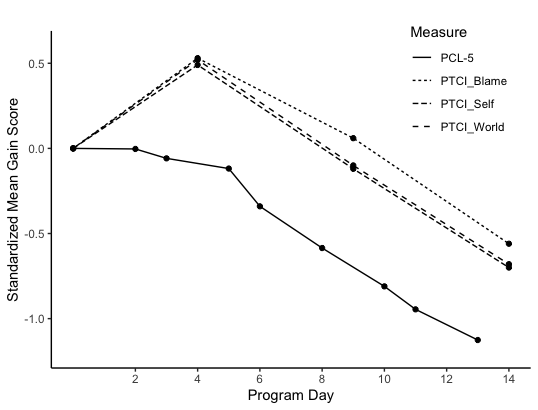


Figure S2. Time trends for PTCI subscales and PCL-5 in 2-week program


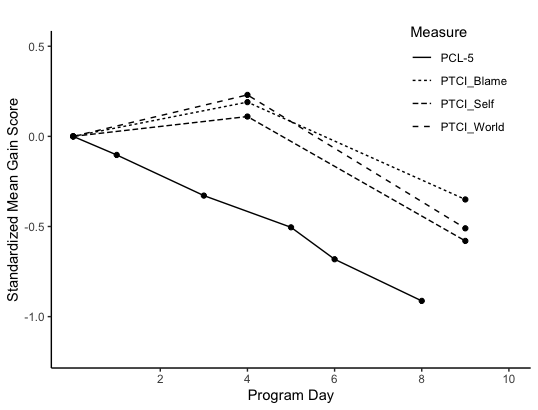

Supplement: Supplementary file 1 — Additional file 1: Table S1. Models Predicting PCL-5 without items 9 and 10. Figure S1. Time trends for PTCI subscales and PCL-5 in 3-week program. Figure S2. Time trends for PTCI subscales and PCL-5 in 2-week program. [file 12888_2022_4296_MOESM1_ESM.docx]
